# Roger’s Homœopathic Guide

**Published:** 1893-11

**Authors:** 


### BOOK NOTICES.

ROGERS' HOMŒOPATHIC GUIDE. By L. D. Rogers, A. M., M. D., and Ida Wright Rogers, M. D. Chicago: People's Health Journal Co., 441 Dearborn Avenue. 1893.

This book is the latest candidate for the favor of the home circle in the treatment of cases of sickness arising suddenly and demanding attention before the doctor can be summoned.

The authors of it are the accomplished and brilliant editors of *The People's Health Journal*, Dr. Rogers and his devoted wife.

The book is decidedly pretentious, being a veritable miniature encyclopædia of diseases and their treatment, and may with perfect propriety be consulted by students and even older practitioners.

It consists of two parts, a part treating of diseases and giving keynote indications for remedies for them, and a part that is all materia medica. This materia medica is rather abbreviated. It might be expanded with benefit. Still, what is given is of sterling value and must be of much assistance to the laity.

From a close inspection of the whole book, we are impressed with the belief that it is very decidedly advanced for the laity, and for that reason we have made the remark that it might be studied with profit by practitioners, especially beginners, and by students.

